# Use of Cocaine

**Published:** 1894-01

**Authors:** 


### Use of Cocaine.

1. Amount of cocaine used must be in proportion to extent of surface it is desired to anæsthetize. In no case should the quantity exceed one grain and three-quarters.

2. Cocaine should never be used in cases of heart disease, pulmonary disease, or in persons of highly nervous temperament.

3. In injecting cocaine, the intradermic method is preferable to the hypodermic. By injecting into, not under mucous membrane or skin, the risk of entering a blood vessel is avoided.

4. During injection the patient should always be in a recumbent position; in operations upon the nose and throat, the head should not be raised until anæsthesia is complete.

5. It is of great importance that cocaine should be pure, since its combinations with certain other alkalies result in poisonous compounds.—*Brooklyn Med. Journal.*

